# Supplementary material for: Empirical methods for controlling false positives and estimating confidence in ChIP-Seq peaks
Source: BMC Bioinformatics. 2008 Dec 5;9:523. doi: 10.1186/1471-2105-9-523 (PMC2628906; doi:10.1186/1471-2105-9-523)
Supplement: Additional File 1 — A variety of html documents from the USeq web site detailing the available applications, their best usage, output file type descriptions, command line menus, etc. [file 1471-2105-9-523-S1.zip › USeqUserGuides/JavaDoc.zip!/JavaDoc/trans/main/SubWindow.html]

SubWindow


|  |  |  |
| --- | --- | --- |
| **Overview** \| **Package** \| **Class** \| **Use** \| **Tree** \| **Deprecated** \| **Index** \| **Help** | |  |
| **PREV CLASS**   **NEXT CLASS** | **FRAMES**    **NO FRAMES**     **All Classes** |  |
| SUMMARY: NESTED \| FIELD \| CONSTR \| METHOD | DETAIL: FIELD \| CONSTR \| METHOD |  |


---


## trans.main Class SubWindow

```
java.lang.Object
  trans.main.SubWindow
```

**All Implemented Interfaces:**: java.io.Serializable

---

``` public class SubWindow extends java.lang.Object implements java.io.Serializable ```

Class to hold information about a subWindow.

**See Also:**: Serialized Form

---

**Constructor Summary**

`SubWindow(int index,
double medianRatio)`


| **Method Summary** | |
| --- | --- |
| `int` | `getIndex()` |
| `double` | `getMedianRatio()` |
| `Oligo[]` | `getOligos()` |
| `void` | `setOligos(Oligo[] oligos)` |

**Methods inherited from class java.lang.Object**

`equals, getClass, hashCode, notify, notifyAll, toString, wait, wait, wait`

**Constructor Detail**

### SubWindow

```
public SubWindow(int index,
                 double medianRatio)
```


**Method Detail**

### getOligos

```
public Oligo[] getOligos()
```

---


### getMedianRatio

```
public double getMedianRatio()
```

---


### setOligos

```
public void setOligos(Oligo[] oligos)
```

---


### getIndex

```
public int getIndex()
```


---


|  |  |  |
| --- | --- | --- |
| **Overview** \| **Package** \| **Class** \| **Use** \| **Tree** \| **Deprecated** \| **Index** \| **Help** | |  |
| **PREV CLASS**   **NEXT CLASS** | **FRAMES**    **NO FRAMES**     **All Classes** |  |
| SUMMARY: NESTED \| FIELD \| CONSTR \| METHOD | DETAIL: FIELD \| CONSTR \| METHOD |  |


---
